# Family Members of Adults with Intellectual Disability Living in Residential Settings: Roles and Collaboration with Professionals. A Review of the Literature

**DOI:** 10.1177/0046958021991301

**Published:** 2021-02-25

**Authors:** Natalie Zambrino, Ingeborg Hedderich

**Affiliations:** 1 Lucerne University of Applied Sciences and Arts, Lucerne, Switzerland; 2 University of Zurich, Zurich, Switzerland

**Keywords:** challenging behavior, family member, intellectual disability, successful collaboration, residential institution

## Abstract

The aim of this article is to review the literature concerning the role of family members of adults with an intellectual disability living in diverse residential settings and their collaboration with residential staff. Whenever the scarce literature on the subject allowed, the focus was laid on family members of persons with additional challenging behavior. Electronic databases, reference screening, and hand search of selected journals was employed to collate literature using key terms such as family members, intellectual disability, and residential setting. By extracting relevant data of the eighteen articles that fulfilled all inclusion criteria, the following 3 main themes with each subthemes were identified inductively: roles of family members after the transition, the effects of the transition on family members, and the collaboration between the family members and professional care staff. This review presents the different roles family members partake and highlights the importance of regular open two-sided communication for collaboration with professional staff to be successful. Practical implementations are discussed and the need for further research in the field is indicated.

**What do we already know about this topic?**
Research has shown the importance of family members as collaborators for professionals in providing comprehensive care for adults with intellectual disability living in residential settings.
**How does this research contribute to the field?**
This literature review combines topic specific articles published between 2000 and 2020 and presents the various roles of family members in comprehensive care for adults with intellectual disability as well as ways for a successful collaboration with professionals.
**What are this research’s implications toward theory, practice, or policy?**
Family members being a part of comprehensive care need to be acknowledged by a broader context to bring along a systemic change for more involvement and support for family members willing to participate in care for adults with intellectual disability living in residential settings.

## Introduction

For persons with intellectual disability leaving the family home is considered a step toward a more independent form of living consistent with societal norms.^1^ In English speaking countries, when reaching young adult age, it became increasingly common for persons with intellectual disability to move out of the parental home to some form of out-of-home residential setting.^2^ Possibilities for independent living and community integration as stated by article 19 of the *Convention on the Rights of Persons with Disabilities* (UN General Assembly, New York, 2006) are therefore necessities. A clear tendency toward more variability, independence, and flexibility in living settings for persons with intellectual disability exists in Switzerland, where this study originates.^3^ Still, the fulfillment of the requirements is an ongoing process with increased numbers for places in residential settings as well as in supported community living between 2011 and 2017 to an almost equal distribution.^3^ Regardless of the living setting, family members represent an important social resource for people with intellectual disability^4,5^ and continue to stay involved after a placement in a residential living setting of any kind, for example, Enosh et al,^6^ and Mailick Seltzer et al.^7^ The service user seems to benefit from this involvement because it may increase the functional achievements of children with developmental disabilities^8^ and can be one factor to secure the proper functioning of care.^1^ Due to well-functioning collaboration between family members and professionals, the former gain advantages as reported in studies from the field of collaboration between professionals and family members of children with intellectual disability in special education or health care. Parents who experience effective collaboration with professionals are more satisfied with the professional support provided and rate their children’s quality of life better than without effective collaboration.^9-11^

The number of publications on the role of family members of *adult* persons with intellectual disability living in residential settings is lower. As Clegg et al^12^ determine, family members often get excluded from research focusing on the transition to adulthood (which, in many cases, entails moving out of the family home). According to them, family members may be seen as superseded by adult services in the pursuit of normalization in areas such as employment, adult role-taking, or independent living. The amount of publications concerning only family members of persons with intellectual disability who show challenging behavior is even scarcer. The term “challenging behavior” refers to a range of behaviors, such as aggression, self-injury, or destructiveness.^13^ The prevalence of challenging behavior in the group of persons with intellectual disability is approximately 10 to 20% for example Allen et al,^14^ and Cooper et al.^15^ while a recent Swiss national study found a prevalence of 28.2% specifically for adults with intellectual disability living in residential institutions.^16^ Current approaches in management of challenging behavior (eg, positive behavior support) pursue a holistic perspective. Thus, the involvement of family members as partners is central.^17,18^ In countries with no formal requirements for residential institutions to collaborate with family members, little is yet known about the potential impact the latter may have on the effectiveness of the care an institutionalized adult with intellectual disability is given.

The aim of the present article is to review the literature on role and collaboration with staff of family members of adults with intellectual disability living in residential settings of all kinds and thus to provide a foundation for future research within this field. To keep the already limited results as broad as possible, a wide definition of the terms “family members” and “residential setting” was used. “Family members” may relate to any kind of family member of closer (eg, parents, siblings, partners) or wider (eg, grandparents, aunts, uncles) range. In terms of “residential setting,” any form of professionally supported living away from the family home was considered (eg, largescale residential living institutions, smaller group homes, supported community settings, permanent stays in psychiatric hospital). The article focuses on the role and involvement of family members in the current living situation of the service user. Therefore, no importance was given to neither former living places (these could be family homes, different residential institutions, hospitals) nor the amount of time that had passed since the move. Most included articles do not specify on the latter factor, but wherever the information is provided, it is specifically stated in the current literature review.

## Methods

**Figure 1.**
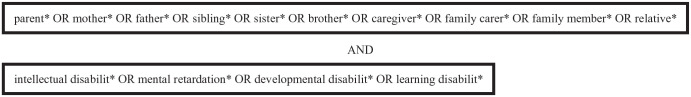
Applied search terms.

**Figure 2.**
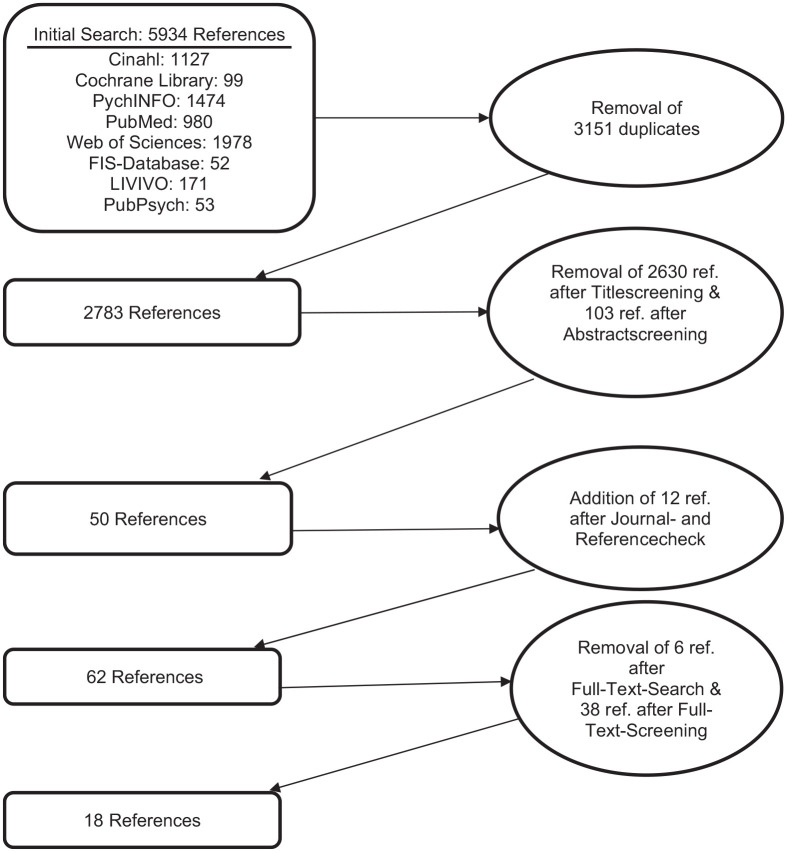
Literature search and review process.

An initial search for articles published between 2000 and 2018 was conducted in May 2019 using the search terms displayed in Figure 1. The following databases covering the areas of psychology, medicine, sociology, and pedagogy were used: Cinahl, Cochrane Library, PsychINFO, PubMED, Web of Sciences Core Collection, FIS Datenbank, LIVIVO, and PubPsych. The last 3 databases are of German origin. There were high numbers of results, therefore, a title search was conducted. Since not all mentioned databases support the use of MeSH terms and broad search results were requested, the search was conducted without it. As parents and siblings are assumed to be the closest family members, these were covered in single search terms. Members of the extended family, such as grandparents or aunts as well as uncles, were summarized in the 3 additional terms: caregiver, family member, and relative. Due to the time-consuming preparation and publication process of this article, an updated search for articles published between 2019 and 2020 was conducted in November 2020. Other than the year of publication, all inclusion criteria as well as the whole literature search and screening were identical. Figure 2 displays the combined results for the 2 search and review processes: After all duplicates were removed, a total of 2783 references was identified. Titles and abstracts were then screened by the first author to ensure the papers fulfilled all inclusion criteria, which were: being written in German or English language, having been published between 2000 and 2020 in peer-reviewed journals, presenting primary research data placing a significant emphasis upon family members of *adult* persons with *intellectual and/or developmental disabilities* living in *residential institutions*. Not mandatory, but of special interest were articles fulfilling all mentioned inclusion criteria with an additional focus on family members of persons with intellectual disability who also show challenging behavior. This reduced the number to 50 references.

After this, hand searching of all issues between 2000 and 2020 of 7 relevant English and German journals as well as a reference checking of the 50 already identified papers took place. This resulted in 12 additional references. During the subsequent full-text search, 6 references could not be found. The remaining 56 references were then full-text-screened by 2 researchers independently in view of their complete fulfillment of the mentioned inclusion criteria. References they did not agree on either including or excluding were discussed until an agreement was reached. A common method for rating agreements between 2 raters places Cohen’s Kappa, which in this case resulted in 0.57, which relates to a moderate agreement.^19^ Although there first was considerable heterogeneity, the discussion process resulted in a total of 18 included articles (Table 1 gives an overview over the included articles). A data extraction sheet was then developed to extract demographic information about the article, method of data collection and analysis, sample consistency and size as well as the relevant content information. By comparing the extracted data and analyzing its similarities and differences, suitable inductive codes, subthemes, and themes were formed. An overview of the extracted information and the developed codes, subthemes, and themes is given in Appendix 1.The themes and subthemes provided the structure for the following results chapter.

**Table 1.** Overview of Included Articles.

Author	Country	Data collection	Sample*	Challenging behavior	Key results
Bigby et al^20^	Australia	Semi-structured interviews every 6 months over a 3-year period	13 Siblings, 17 staff members	No	Siblings play a crucial role in securing the wellbeing of the service user.
					Sibling-staff relationships are dynamic.
Bonell et al^25^	UK	Semi-structured interviews	11 Parents, 3 siblings, 2 partner	Yes, partially	Family members (FM) stay involved in diverse roles after transition to out-of-area specialist hospitals.
					FM often feel sidelined by professionals.
Bright et al^28^	UK	Semi-structured interviews	5 Mothers, one aunt, one sister	No	Most concerns of FM are about “little things.”
					Importance of open and honest communication between FM and staff.
Cernikovsky^21^	USA	Semi-structured interviews	4 Mothers, 2 fathers	Yes, partially	The parental role changes after the move. Parents keep on maintaining contact to the service user and there is a diminishing level of involvement in overall care.
					Emotional support is a central task for parents.
					There are positive and negative effects of the move for parents: for example, relieve about the decreased role they fulfill and feeling doubtful and/or guilty.
Chase and McGill^31^	UK	Semi-structured interviews	5 Sisters	Yes	All participants stay involved in the life of the service user and see themselves as the next-in-line carer.
					A need for ongoing support not only for parents, but also siblings after the service user reaches adulthood is stated.
Doody^26^	Ireland	Interviews	7 Sisters, one mother, one brother, one niece, one brother-in-law	No	The move from psychiatric institution to community-based institution is experienced positively by FM and brings along more open and transparent systems and thus higher involvement of FM.
					A team approach to care and good communication is seen as key for a quality collaboration.
Enosh et al^6^	Israel	The Family Environment Scale and The Parental Involvement Scale/Interviews	216 Parents	No	Parental behavior involvement is dependent on the service user’s gender, age at placement and intertwined with the other forms of involvement (cognitive and emotional) along the Parental Involvement Scale.
Jansen et al^36^	Nether-lands	Self-administered logbook entries over a 12 months period	3 Mothers	No	Contacts between mothers and professionals mostly have the function to exchange information and mostly cover issues related to health.
					A majority of these contacts is experienced positively.
Li^33^	Hong Kong	Semi-structured interviews	6 Sibling advocates	No	Advocating for more service provision to the service user, improve service quality and facilitate communication between professionals and FM were the main tasks of siblings functioning as advocates.
McKenzie et al^29^	UK	Semi-structured interviews	8 Family members	Yes	FM appreciate a strong value base and high levels of knowledge and skills in professionals for a successful positive behavior support and thus a decrease in challenging behavior.
					FM maintain a central role in the life of the service user and want to be involved.
					Involvement of FM may be beneficial for the service user and FM themselves.
Mailick Seltzer et al^7^	USA	Interviews every 18 months over a 12-year period	Wave: 117 mothers	No	FM provide less formal care after the move, but are central for emotional support and social participation.
			Wave: 64 mothers		Mother’s health is on average more stable or increase when the adult child with ID left family home.
			Wave: 26 siblings		Mothers worries about the service user decline over time, so did contact to residential staff.
Rimmerman and Chen^24^	Israel	2 Structured interviews (questionnaires) in a 6 months period	63 Parents and 67 siblings	No	Visits to the service user seem to be related to the relationship quality.
					For parents, habit and duty further play a role, while for siblings perceived control does so.
Rimmerman and Muraver^35^	Israel	Questionnaires	160 Mothers	No	Mothers of adults with ID living at home seem to be confronted with fewer undesired live events than mothers of service users in residential institutions.
Schwartz^22^	Israel	Questionnaires	39 Fathers, 32 Mothers	No	Parental involvement is expressed by frequent visits and participation in social activities. Parents express a high degree of satisfaction with the extent of contact with the service user and staff.
					20% of the parents perceive a full partnership between themselves and residential staff in the service user’s care.
Tozer and Atkin^30^	UK	Qualitative interviews	14 Sisters and 7 brothers of persons with autism and learning disability, 12 professionals, 12 persons with autism and learning disability	No	Siblings feel a commitment for the service user and continue to be involved in his/her life.
					Experiences in collaboration with professionals are mixed. Some describe positive relationships others feel excluded.
Walker and Hutchinson^32^	Australia	Semi-structured in depth interviews	9 Aging parents	No	Parents continue to see themselves as primary care-givers and offered a high level of support to the service user, resulting in strong feelings of purpose and continuity of a valued role.
Williamson and Meddings^27^	UK	Semi-structured interviews	3 Mothers, 1 father	Yes, partially	The transition to an institution brings along role uncertainties for parents. Roles may change over time.
					Parents value collaboration with professionals.
					Negative experiences in collaboration are shared, for example, poor exchange in information, no possibility to speak up in meetings with professionals.
Wong and Wong^23^	Hong Kong	Focus group-discussions	18 Mothers, 7 fathers	Yes, partially	Parents maintain frequent visits to the service user after the transition and take part in residential activities, less in formal care.
					A majority of participants lack knowledge on their child’s disability.

*Note*. *Sample numbers accord to family members of adult persons with intellectual and/or developmental disabilities living in residential institutions. In 3 cases and with clearly distinguishable results, not the complete original sample was used due to part of it not fulfilling all sampling criteria.

## Results

Table 1 describes key characteristics of all 18 included articles. Six studies originate in the UK, 4 in Israel, 2 each in Australia, the US, and Hong-Kong and 1 each in Ireland and the Netherlands.

In total, 3 main themes with respective subthemes were identified arising from the data extraction: Roles of family members after the service user’s transition to the residential setting, effects of the new role on family members, and collaboration between family members and residential staff. Results may concern a variety of family members but an unsurprising focus on parents and siblings emerged, since these represent the closest relatives in most cases. Therefore, unless specifically mentioned otherwise, the following results refer to these 2 groups.

Generally, after the service user’s transition to a residential setting, the family members maintain contact with various frequencies^7,20,21^ and periodic visits.^22,23^ Rimmerman and Chen^24^ found, that for siblings the amount of visits to the service user is related to the relationship quality and perceived difficulty respectively perceived control. For parents visits are more commonly related to habit and duty.

Overall, contact with service users living in residential services for people with intellectual disability rather diminished after the move.^20,21^ For persons with intellectual disability and severe challenging behavior who are being cared for in psychiatric hospitals, Bonell et al^25^ found no contact with relatives for some of the interviewed families due to damaged relationships or for the service user to get better. Doody^26^ reported increasing levels of contact after moving out of psychiatric hospital care to a community-based disability service.

### Roles of Family Members After The Service User’s Transition to a Residential Setting

Cernikovsky^21^ conducted semi-structured interviews with 4 mothers and 2 fathers of adult persons with intellectual disability living in community group housing. They describe their parenting role as ongoing, but changing after the individual with intellectual disability moved into the residential setting. This was confirmed by Doody,^26^ who interviewed 11 family members including 1 niece and 1 brother-in law, whose relative with intellectual disability moved from a psychiatric hospital to a community-based disability service. To interviewed family members, accepting the change in role concerning one’s own involvement in care and the planning of care for the relative with intellectual disability is central and valued positively by most, this study found. After having interviewed 4 parents, Williamson and Meddings^27^ state that transitioning to an institutional setting brings along uncertainties about the definition of the new roles. Some parents said that they were strongly involved as advocates in the beginning, but later took on more distant roles. A diminishing level of parental involvement is also stated by Cernikovsky.^21^

Overall, many articles describe the family members’ continued involvement in one way or another in the service user’s life after the transition to a residential setting.^20,21,28,29^ for positive behavior support,^30-32^ In the following, the different and not mutually exclusive roles that emerged from the articles are outlined.

#### Formal roles

Bigby et al^20^ interviewed 14 siblings every 6 months over a duration of 3 years after the sibling with intellectual disability moved to a residential institution. Some siblings described taking on different formal roles, for example, health guardian or administrator, which involve clearly delineated responsibilities. However, most siblings simply described their role as next-of-kin, which in some cases led to uncertainties regarding responsibilities—especially so in decision-making with staff members. Mailick Seltzer et al^7^ as well as Walker and Hutchinson^32^ reported mothers to help their adult children with their finances. No other articles describe formal roles.

#### Secure wellbeing of service user

Besides formal roles, family members described other tasks they perform to secure the service user’s care and wellbeing. One study^31^ found siblings to continue being actively involved in the care of the service user. They described themselves as next-in-line carers and reported feelings of pressure due to imminent additional responsibilities for the service user. Different articles reported functions, that can be summarized as roles of advocates: communicating with staff on behalf of the service user,^28^ Voicing their concerns about the care the service user is given^25,32,33^ and demanding better service provision as well as noticing changes in the service user’s behavior that might indicate that they were unsatisfied with the services.^28,32^

Some parents described having been heavily involved as advocates after the transition at first, but said that the level of involvement decreased over time.^27^ Li^33^, who specifically interviewed 6 Hong Kong based sibling advocates, found that all participants were invited by the residential service to participate as advocates. The interviewed siblings experienced positive effects through their advocacy work. Namely, they felt more open and positive and they also expressed the wish for training provision and assistance for new family members taking up advocacy tasks.

Other parents who were interviewed by Cernikovsky^21^ mentioned that facilitating the communication between the service user and staff was not unidirectional. In some cases, parents had to step in for residential staff and translate expectations toward the service user. Some even described situations in which they had to alter or override the actions of their children, mostly to protect them from consequences of their own decisions especially in aspects concerning their health or in situations which included challenging behavior. On the other hand, for siblings on the autism spectrum, Tozer and Atkin^30^ describe feelings of protectiveness toward their brother or sister in situations with residential staff. Similarly, McKenzie et al^29^ found, that family members felt the need to protect the service user with challenging behavior from inappropriate, sometimes even abusive treatments, such as not treating them as adults or overprescribing medication.

A topic that recurs in many articles is that of thoughts about the service user’s future. Parents are well aware that in most cases their children with intellectual disability would outlive them. Thus they see the importance of ensuring that someone would replace them.^20,30,32^ For some families, this topic evoked feelings of guilt and concern.^28^ As 2 articles demonstrate, siblings play a central role in securing the future. As parents grow older, siblings become aware of how their involvement gradually increases and feel a need to discuss their future involvement with the whole family. Knowing about the importance of this topic, siblings wish for proactive future planning initiated by professionals because they are often unsure about how to start a dialog between professionals, parents, and themselves.^30^ Similarly, a Hong Kong based study^23^ found that parents who are still involved in different caring tasks wish for siblings to be part of future care, but have never initiated a dialog with them, thinking that they had to face the burden themselves.

As part of ensuring the service user’s wellbeing, family members show interest in the care their relative with intellectual disability receives. Bigby et al^20^ found siblings to “keep an eye on things” and make sure they are well-informed about aspects related to the care of their brother or sister with intellectual disability. Thereby, they want to check the quality of care and pre-empt any upcoming issues. Also, Li^33^ found siblings to be willing to enhance the care the service user benefits from and that they are thus interested in staying informed about care-related activities.

#### Supplement formal care: Emotional and social support

Mailick Seltzer et al,^7^ who accompanied mothers during and after the relocation process from family home to residential institution state an unsurprising significant decrease in hands-on care from pre-transition to each of the 3 post-transition data collection points. In the study by Wong and Wong,^23^ parents preferred visiting their children in the residential institution over taking them home due to difficulties in coping with possibly physically demanding caring tasks such as lifting the son or daughter for example. As expected, rather than lending support to formal care, family members took on other tasks. Providing emotional support was found to be central in 3 studies.^7,20,21^

Enosh et al^6^ examined a model predicting parental behavioral involvement in 216 parents of adults with intellectual disability placed in institutions in Israel using the Parental Involvement Scale.^34^ This scale measures 3 factors of parental involvement: cognitive involvement, emotional involvement, and behavioral involvement. Enosh et al^6^ found different factors that impact parental involvement: the adult child’s sex, age, and distance between the institution and the family home. Parents of a girl with intellectual disability had higher scores in emotional involvement and lower scores in cognitive involvement. As the child’s age increases, the parents’ cognitive involvement decreases and the further the distance between the residential institution and the parents’ home, the lower the level of their behavioral involvement is. Furthermore, they found that emotional involvement did not have an impact on behavioral involvement, but cognitive involvement did with a small effect size. Also, overall stress at the parental home seems to decrease all forms of involvement.

Other tasks family members regularly took on are to accompany persons with intellectual disability in social activities inside or outside their residential institution or to facilitate their participation in the community with or without taking them home for a visit and thus giving relief to residential staff.^7,20,23,25,32^

Other ways parents complemented the formal care provided by the residential institution’s staff were going shopping with the service user^7,32^ or payment for additional services, such as counseling,^20^ arranging and attending medical care, and partly managing the food intake by delivering frozen meals to the service user.^32^ Bigby et al^20^ found siblings to be the ones wanting to break any kind of bad news to the service user instead of letting them know via residential staff. Walker and Hutchinson^32^ found the 9 interviewed parents to take on such responsibilities to alleviate some of the responsibility for residential staff or because the residential providers’ budget was limited.

McKenzie et al^29^ found that all 8 of the interviewed parents of service users with challenging behavior want to be involved in their child’s care. Also the interviewed point out their profound knowledge of their son or daughter and they are aware of their valuable expertise when it comes to managing the challenging behavior they display.

### Effects of New Roles on Family Members

The change in roles that family members took on after their relative with intellectual disability left the family home effected the lives of both the service user’s parents and siblings and triggered diverse feelings. The following 3 sections portray these effects.

#### Positive effects

Different positive effects were identified in the included articles. Mailick Seltzer et al^7^ checked health ratings of mothers at different times before and after the person with intellectual disability moved to residential living. Those findings were then compared to health ratings of mothers whose relative with intellectual disability still co-resided with them. They found the health of mothers whose children with intellectual disability had moved out to be on average more stable or even increased over the study period. Cernikovsky^21^ found mainly positive effects on parents after their child with intellectual disability moved out; 1 mother described the effect as “getting her life back.” Furthermore, she spoke about how she experienced decreased stress levels and increased independence since her son moved out as she no longer had to balance a full time job with her son’s care needs. The mother further talked about the positive effect her newly gained independence had on other personal relationships, such as her marriage. All the interviewed persons in this study^21^ said that they discovered new pleasurable interests, especially travelling as an activity they would not have been able to do whilst their child was still living at home. Chase and McGill^31^ concluded a significant impact on the life of the siblings they interviewed and reported a positive outlook. The siblings developed personal character traits such as empathy, patience, and acceptance and reported a positive influence on their career choices. Walker and Hutchinson^32^ concluded the 9 parents they interviewed, to derive purpose and pride from their current parenting role.

Several articles described family members’ experience of positive feelings and reactions in connection to the individual with intellectual disability leaving the family home. Cernikovsky^21^ and Doody^26^ found positive feelings in many parents they interviewed who told about finding their children with higher levels of maturity and independence after the move. Mailick Seltzer et al^7^ report a significantly increased satisfaction in mothers about the amount of contact they had to their adult children with intellectual disability over time. Parents feel relief and satisfaction because residential staff assume responsibility for formal care and thereby take pressure off the parents’ role^26,29^ and were reassured as soon as the service user had settled in and was happy about the new life.^30^

#### Negative effects

Besides positive effects, the new roles family members took on after their son or daughter with intellectual disability left the family home also brought some negative effects. Some family members were confronted with a sense of social stigmatization. For example, some parents were reluctant to tell other people about visiting their daughter or son in a long-stay psychiatric institution, which were also referred to as “mad houses.”^23,26^ In their interview study on 7 female family members, Bright et al^28^ found that the participants, who were still highly involved, described a strong emotional component to their experience. They reported that conflicts between their support in care and other tasks (such as work) still existed and left them with feelings of guilt and anxiety. Rimmerman and Muraver,^35^ who compared mothers of offspring with intellectual disability living at home to mothers of offspring living out-of-home with regard to undesired life events. They found mothers whose offspring still lived at home to be confronted with fewer undesired life events than their counterpart.

Family members reported worries about the service user and the changed situation in general. This was reinforced by different factors, such as insufficient communication from residential staff or general dissatisfaction with the service their child with intellectual disability received.^23,28,29,36^ Mailick Seltzer et al^7^ found mothers’ worries to significantly decline in the long term and finally drop below the level of the comparison group consisting of mothers who continued to live with their adult child with intellectual disability. Commonly reported are feelings of guilt and doubt, for example, for not assuming the same level of responsibility they used to.^21,27,28,30^ Wong and Wong^23^ found some parents confronted with self-blame for their child’s disability and for some families, Bonell et al^25^ report feelings of shame for the challenging behavior the service user displays. Tozer and Atkin^30^ found siblings to feel torn between their involvement in their sister’s or brother’s life and other commitments such as work or their own family. Related to residential staff, some family members feel excluded from discussions and future planning^30^ and disempowered by staff taking over control of the information flow.^21,25^

### Collaboration Between Family Members and Residential Staff

When a person with intellectual disability decides to leave the family home to live in a residential institution, responsibilities shift, and new relationships emerge not only between the service user and residential staff but also between residential staff and family members.^7,26^ Family members have different expectations. Some anticipate residential staff to become a form of parental substitute and later realize that staff would not take over a parental role but create a new form of professional relationship leaving the family responsibilities with the family members.^21^ Thus, the relationship and collaboration between family members and staff gains importance and needs to be negotiated. Bigby et al^20^ found the nature of this relationship and contact to be dynamic—at times tense but predominantly easy—and characterized by respect, successful communication and the feeling of “being in a team with staff.” Tozer and Atkins^30^ came to similar findings: Siblings were aware of their clearly proactive role in communication with staff and appreciated institutions that made an effort to include the family members into the service user’s life. Schwartz^22^ on the other hand found only about one fifth of the parents in her sample to perceive themselves as full partners to residential staff. Bigby et al^20^ found tension to rise within the relationships on occasions that included the loss of trust in staff or communication breakdown. Generally, contact shifted over time with more pronounced change right after the move^7^ and after incidents such as changes to the care of the service user or accidents.^36^ Rimmerman and Chen^24^ found different variables influencing the frequency of contact between family members and staff, such as the amount of visits to the service user, his or her mental and psychiatric functioning levels and self-controllability. For parents and for siblings, higher frequency of hosting was shown to lead to more contact to staff and being of Asian or African origin was shown to less contact to staff. However, the nature of the relationship was negotiated between siblings and staff and due to its unscripted character, it was able to adjust to changes in surroundings.

#### Beneficial aspects for successful collaboration

The articles included in this review contain different aspects that are beneficial to collaboration between family members and residential staff. Williamson and Meddings^27^ interviewed family members and were told by some that the process of letting the relative with intellectual disability go after he or she moved out of the family home was facilitated by being reassured that the service user receives good quality care. But most importantly, regular, open, and honest communication was seen as the key factor to good collaboration and to overall positive experiences for all persons involved and therefore, had a unifying effect on all relationships.^20,28,36^ All the participants in Williamson and Meddings^27^ study generally reported on positive staff attributes and approaches, for example, having experienced staff’s warmth, friendliness, compassion, empathy, experience, and honesty. McKenzie et al^29^ describe family members to value knowledgeable and competent staff with a strong base of values toward the service user and his or her support. Bright et al^28^ found family members to recognize the difficulties of the staff’s work and made sure to provide them with positive feedback. Similarly, McKenzie et al^29^ presented results about successful collaboration between parents of persons with intellectual disability and challenging behavior and staff. They told about professionals willing to collaborate after having made the experience that challenging behavior management suggested by the parents had a positive effect on the service user. Another positive experience shared by these parents was staff not only being there for the service user, but also for the family members when dealing with their own emotions and challenges.

#### Obstructive aspects for successful collaboration

Negative experiences were diverse, but mostly somehow connected to communication issues. Examples were total communication breakdowns,^20^ breaking promises, and lack of communication in the professional team. More example were not listening to family members,^36^ low responsiveness, staff taking family member’s concerns too personal and thus unsettle them^28^ as well as general miscommunication.^21^ Some family members told about insecurities about whom they could turn to^25^ or how they could initiate a discussion about topics they felt were important to the service user.^30^ Another central concern was that family members felt like they were not being listened to or that their concerns were not taken seriously.^28,30,36^ Some also mentioned similar experiences in connection with meetings, such as not being invited to team meetings or not getting the possibility to speak in meetings.^25,27^ Some family members reported lacking information about the service user’s care to cause worries and a loss of trust.^25,36^ On the other hand, Wong and Wong^23^ found a majority of their sample of parents living in Hong Kong to lack information and knowledge about their child’s condition and developmental possibilities.

Schwartz^22^ states rather high numbers for Israeli parents who have never met with a staff member (31%) or have met once or twice (38%) since their adult children left home (mean length of stay 3.66 years, SD = 1.46). Tozer and Atkin^30^ support these findings and report that only few of the siblings they interviewed had met their sibling’s care manager, unless there had been a crisis. More, Schwartz^22^ found low ratings of partnership between residential staff and family members. Twenty percent of parents perceived a full partnership between themselves and the professionals caring for the service user.

Tozer and Atkin^30^ reported about one third of the siblings who participated in the study to feel unfairly judged by the staff.

For many family members, communication issues led to a loss of trust or a general mistrust.^28,30,36^ For some, this meant adjusting their controlling activities, which resulted in friction with staff.^20^

Parents of service users with challenging behaviors acknowledged staff to be influenced by a wider system than the residential unit alone and the thus emerging difficulties. They declared a need for collaboration in the overall system to offer comprehensive care, especially when challenging behavior is present.^29^

Cernikovsky^21^ found 1 parent not confident with his role with regard to the level of passiveness since he expected even greater support from staff. Furthermore, he found some parents who expected the relationship between the service user and the staff to exceed a purely professional level, not to be satisfied with the support their child receives.

Finally, family members mentioned that organizational changes, high staff turnover-rates or shortage of staff hinder smooth communication and collaboration with staff.^20,21,25,30^

## Discussion

All considered articles reported a change in the family members’ roles after an individual with intellectual disability transitioned to a residential setting. One more consensus is that involvement in the life of the service user decreases after the move and, sometimes after a first peak directly after the transition, contact to the relative with intellectual disability does likewise but becomes stable in the long term. This is supported by other publications with a comparable focus.^4,37,38^ For an overview of collaboration with support services, if the service user is co-residing

Considering the roles that were taken by the family members after the move, a high diversity can be identified with a focus on tasks aside from formal care. This was to be expected since formal care tasks are 1 central part of the residential living offer. Parents and siblings complement formal care in different ways such as joining their relative for social activities, offering them community access, taking on formal roles and importantly, giving emotional support.^7,20,21^ With this division in tasks and parents complementing formal care, a regular dialog between family members and residential staff appears relevant and ultimately benefits the service user.

Although family members play a minor role in formal care, they are still interested in the care their relative with intellectual disability receives and feel the need to “keep an eye on it.”^20^ This is reinforced when communication issues between residential staff and family members arise, for example, when communication breaks down or staff is perceived to be untrustworthy. On a basis of good communication and mutual trust, family members may decrease care monitoring and take over supplementary tasks to enrich rather personal parts of the service user’s life, such as providing possibilities for community access or offering emotional support when needed.

Similar to Baker and Blacher,^1^ who reported advantages and disadvantages for family members of children and adults with intellectual disability emerging from residential placement, the articles included in this review saw family members to be confronted with a variety of emotions after the transition. They feel positive about their adult children’s or siblings’ growth in personality or the increase of independence and freedom in their own lives. On the other hand, family members experience negative emotions about the service user’s treatment, they can feel guilty or have doubts, and they sometimes struggle to balance their involvement in care with other demands all of which leads to them being at risk of experiencing exacerbated stress.^38^

The relationships between family members and residential staff are of a negotiated nature and get easily disturbed by unfortunate events or communication issues. Generally, regular dialog between family members and professionals is the key to successful collaboration.^12,38^ If this is nonexistent, many support services tend to increase rather than decrease the burden of care.^38^ Family members then tend to grow mistrust which in turn leads to them feeling a more pressing need to monitor the formal care. Open and honest communication has a unifying effect which is instrumental to mutual decision-making, especially in future planning, an often emotional topic for many groups of people involved. Regular possibilities for both formal and informal exchanges are recommended, such as giving family members the opportunity to voice their concerns in scheduled staff meetings or taking the time for an impromptu conversation with them when they pick up or bring back the service user after a visit at home.

In Switzerland﻿, where this study originates, there is a background of family members complementing staff’s daily work by resuming responsibilities for the service user and also helping staff by sharing their knowledge about the service user with them.^29^ Therefore, it is surprising that there is yet little embedded participation structurally established in care programs in residential institutions. Li^33^ suggests building an advocates model for family members that considers individual institutional particularities and needs. This may constitute a possibility of how to not only strengthen the relationship between professionals and family members, but also how to accredit the role of the latter with the importance it deserves. Other possibilities to sensibly integrate family members into care include diaries filled out by both parties^12^ or more recent forms of digitalized ways of exchange.

The perception of service users by family members and professionals can differ considerably due to their unique history and experience with them. Clegg et al^12^ exemplify these divergent views. They found that staff working with persons with intellectual disability and challenging behavior are rather future-oriented and that they prioritize adult autonomy above dependent partnership. Meanwhile, parents and other family members are more likely to strive for continuity based on past experiences. Simultaneously, they found, that staff working with persons, whose challenging behavior remains more likely adopted an ahistorical approach, which can be seen to support the comprehensive approach suggested in the present article. Finding a fruitful way for family members and professionals to work together without disregarding the autonomy and self-efficacy of the individual with intellectual disability may thus help to reduce challenging behavior.

Research has shown that compared to the general population, family carers co-residing with an adult with intellectual disability exhibit poorer health outcomes. Inevitably, a need for further support is outlined.^39^ Equally, the demand for accessible support systems for persons with a relative with intellectual disability living in a residential institution that help them with emerging issues in their changing role or in collaboration with professionals is made.^31^ This needs to be recognized, when attempting to include family members into the comprehensive care program of the service user.^40^

### Limitations

This review contains different limitations. Firstly, the term “residential institution” was defined as a deliberately broad term due to scarcity of existing literature. This resulted in a high diversity of residential placements in the here reviewed 18 articles, including everything from long-term psychiatric placements to largescale residential living institutions and also smaller forms of group-based community living. The same applies to different lengths of stay in the residential institution. When interpreting the results, one also has to keep in mind that international social systems and forms of residential institutions as part thereof are highly diverse and dependent on a country’s political system, its culture and its history. Additionally, persons with different forms and levels of intellectual disability and displaying various forms of challenging behavior may be in need of differing support forms, which has not been taken into account in this review. All of these factors may affect involvement of family members and considering them for future research in the field might be helpful. Secondly, besides a shortfall in specification, there is the matter of potentially disregarded articles. The present review may have missed articles that are relevant to the topic due to the application of title only search, the exclusion of gray literature, the selection of the used databases, and the search for articles written in English and German language only. Thirdly, due to limited resources, the initial literature search as well as the title- and abstract-screening was solely performed by the first author. To balance this out, a second independent researcher engaged in the full text-screening to ensure the fulfillment of all inclusion criteria for the finally included articles.

## Conclusion

Irrespective of the living setting, many family members stay involved in the life and care of service users with intellectual disability and fulfill various role, mainly complementing residential care. In doing so, family members constitute knowledgeable and experienced resources when it comes to dealing with the service user and managing potentially present challenging behavior.

Given that the service user approves of collaboration between family members and staff, these resources can be shared and an authentic, collaborative partnership between both parties can be established. A prerequisite is, however, that structurally embedded participation possibilities are created that allow for regular, open, bilateral exchange of information, opinion, or expectations to take place and to prevent friction between those involved. Family members are an integral part of comprehensive care and should be recognized as such. This needs to be acknowledged by a broader context to bring along a systemic change. Involvement for family members willing to participate in the care for institutionalized adults with intellectual disability needs to be possible and easily initiated. Also, adequate support systems for these family members are required. When establishing these structures, taking into account experiences from countries where such collaboration is a formal requirement, can be beneficial.

There is a need for further research into what is required for and expected of successful collaboration from both parties, family members, and professional care staff. Also, the implications of this kind of collaboration for adult service users should be studied in countries that have yet little experience in comprehensive care approaches that let family members of persons with intellectual disability participate.

**Appendix 1.** Themes Development.

Extraction item/Themes	Subthemes (if applicable)	Codes (if applicable)
Demografic information		
Authors		
Year of publication		
Country of origin		
Methods		
Quantitative and/or qualitative methods		
Method of data collection and analysis		
Sample		
Sample description		
Sample size		
Age of persons with disability	Adult	
	Specific age mentioned	
Type of disability	Intellectual and/or developmental disability	
	Additional disability	
Type of residential living institution	Unspecified group home	
	Community housing	
	Hospitals/psychiatric clinic	
Additional focus on challenging behavior of adults with intellectual and/or developmental disability	Yes. What type?	
	No	
Content information		
Roles of family members after transition	Formal roles	Administrator
		Support with finances
		Health guardian
	Secure wellbeing of service user	Different advocates tasks
		Protect service user
		Secure the future
		Monitor care
	Supplement formal care: Emotional and social support	Emotional support
		Accompanying social activities
		Other support
Effects of role on family members	Positive effects	Increased physical health
		Increased mental health
		More time
		Effects on other relationships
		Positive feelings
	Negative effects	Social stigmatization
		Conflicts with other responsibilities
		Increased undesired live events
		Negative feelings
Collaboration between family members and residential institution’s staff members	Beneficial aspects for a successful collaboration	Conscious letting go
		Positive aspects related to communication
		Positive staff attributes
	Obstructive aspects for a successful collaboration	Negative aspects related to communication
		Lack of personal meetings
		Unmet expectations of partnership
		Negative effects of broader system
		Organizational/staff changes
Additional relevant information	Relevant general and/or divers specific information aside above mentioned subthemes	

## References

[1] Baker BL Blacher J. For better or worse? Impact of residential placement on families. Ment Retard. 2002;40(1):1-13. 11806729 10.1352/0047-6765(2002)040<0001:FBOWIO>2.0.CO;2

[2] Braddock D Emerson E Felce D Stancliffe RJ. The living circumstances of children and adults with MR/DD in the United States, Canada, England and Wales, and Australia. Ment Retard Dev Disabil Res Rev. 2001;7:115-121. 11389566 10.1002/mrdd.1016

[3] Fritschi T Von Bergen M Müller F, et al. Bestandesaufnahme des Wohnangebots für Menschen mit Behinderungen. Schlussbericht zuhanden des BSV. Berner Fachhochschule; 2019.

[4] Alich S. Angehörige erwachsener Menschen mit Behinderung. Ein Problemaufriss: empirisch-exemplarische Darstellung zur Lebenslage Angehöriger von Menschen mit Behinderung in Einrichtungen der Behindertenhilfe. Lit; 2011.

[5] Blacher J Baker BL. Toward meaningful family involvement in out-of-home placement. Ment Retard. 1992;30(1):35-43. 1556937

[6] *Enosh G Rimmerman A Hozmi B Araten-Bergmann T. Israeli parents’ involvement with their adult children with intellectual disabilities after placement in institutional care: a national study. Int J Rehabil Res. 2007;30(4):351-356. 17975458 10.1097/MRR.0b013e3282f144eb

[7] *Mailick Seltzer M Wyngaarden Krauss M Hong J Orsmond GI. Continuity or discontinuity of family involvement following residential transitions of adults who have mental retardation. Ment Retard. 2001;39(3):181-194. 11419997 10.1352/0047-6765(2001)039<0181:CODOFI>2.0.CO;2

[8] Moxley DP Raider MC Cohen SN. Specifying and facilitating family involvement in services to persons with developmental disabilities. Child Adolesc Social Work. 1989;6(4):301-312.

[9] Blue-Banning M Summers JA Franklang HC Lord Nelson L Beegle G. Dimensions of family and professional partnerships: constructive guidelines for collaboration. Except Child. 2004;70(2):167-184.

[10] De Greeter KI Poppes P Vlaskamp C. Parents as experts: the position of parents of children with profound multiple disabilities. Child Care Health Dev. 2002;28(6):443-453. 12568473 10.1046/j.1365-2214.2002.00294.x

[11] Dunst CJ Trivette CM Hamby DW. Meta-analysis of family-centered help giving practices research. Ment Retard Dev Disabil Res Rev. 2007;13:370-378. 17979208 10.1002/mrdd.20176

[12] Clegg J Sheard C Cahill J. Severe intellectual disability and transition to adulthood. Br J Med Psychol. 2001;74:151-166. 11453168

[13] Emerson E. Challenging Behaviour. Analysis and Intervention in People with Intellectual Disabilities. Cambridge University Press; 1995.

[14] Allen DG Lowe K Moore K Brophy S. Predictors, costs and characteristics of out of area placement for people with intellectual disability and challenging behaviour. J Intellect Disabil Res. 2007;51:409-416. 17493024 10.1111/j.1365-2788.2006.00877.x

[15] Cooper SA Smiley E Jackson A, et al. Adults with intellectual disabilities: prevalence, incidence and remission of aggressive behaviour and related factors. J Intellect Disabil Res. 2009;53:217-232. 19178617 10.1111/j.1365-2788.2008.01127.x

[16] Calabrese S Hassler B Bueschi E Lustenberger N Schicka M. Merkmale spezialisierter Institutionen für Menschen mit kognitiven Beeinträchtigungen und herausfordernden Verhaltensweisen. VHN. 2019;88(1):e1-e15.

[17] Brown J Nolan M Davies S. Who’s the expert? Redefining lay and professional relationships. In: Nolan M Davies S Grant G, eds. Working with Older People and Their Families. Open University; 2001:19-32.

[18] Hastings RP Allen D Baker P, et al. A conceptual framework for understanding why challenging behaviours occur in people with developmental disabilities. Int J Pos Behav Sup. 2013;3:5-13.

[19] Landis JR Koch GG. The measurement of observer agreement for categorical data. Biometrics. 1977;35:159-174. 843571

[20] *Bigby C Webber R Bowers B. Sibling roles in the lives of older group home residents with intellectual disability: working with staff to safeguard wellbeing. Aust Soc Work. 2015;68(4):453-468.

[21] *Cernikovsky DT. Parental Attitudes Toward Involvement in the Lives of Adults with Intellectual and Developmental Disabilities Following Residential Transition. PhD Thesis. The State University of New Jersey, USA; 2015.

[22] *Schwartz C. Parental involvement in residential care and perceptions of their offspring’s life satisfaction in residential facilities for adults with intellectual disabilities. J Intellect Dev Disabil. 2005;30(3):146-155.

[23] *Wong SY Wong TKS. An exploratory study on needs of parents of adults with a severe learning disability in a residential setting. Issues Ment Health Nurs. 2009;24(8):795-811. 13129754

[24] *Rimmerman A Chen A. Predicting supportive behavior of parents and siblings to a family member with intellectual disability living in institutional care. J Soc Work Disabil Rehabil. 2012;11:143-165. 22900737 10.1080/1536710X.2012.703927

[25] *Bonell S Ali A Hall I Chinn D Patkas I. People with intellectual disabilities in out-of area specialist hospitals: what do families think? J Appl Res Intellect Disabil. 2011;24:389-397.

[26] *Doody O. Families’ views on their relatives with intellectual disability moving from a long-stay psychiatric institution to a community-based intellectual disability service: an Irish context. Br J Learn Disabil. 2011;40:46-54.

[27] *Williamson H Meddings S. Exploring family members’ experiences of the Assessment and Treatment Unit supporting their relative. Br J Learn Disabil. 2018;46:233-240.

[28] *Bright N Hutchinson N Oakes P Marsland D. Families’ experiences of raising concerns in health care services: an interpretative phenomenological analysis. J Appl Res Intellect Disabil. 2018;31:405-412. 28994488 10.1111/jar.12419

[29] *McKenzie K Mayer C Whelan KJ McNall A Noone S Chaplin J. The views of carers about support for their family member with an intellectual disability: with a focus on positive behavioural approaches. Health Soc Care Community. 2018;26:e56-e63. 28695628 10.1111/hsc.12475

[30] *Tozer R Atkin K. ‘Recognized, valued and supported’? The experiences of adult siblings of people with autism plus learning disability. J Appl Res Intellect Disabil. 2015;28:341-351. 25753367 10.1111/jar.12145

[31] *Chase J McGill P. The sibling’s perspective: experiences of having a sibling with a learning disability and behaviour described as challenging. Tizard Learn Disabil Rev. 2019;24(3):138-146.

[32] *Walker R Hutchinson C. Care-giving dynamics and futures planning among ageing parents of adult offspring with intellectual disability. Ageing Soc. 2019;39:1512-1527.

[33] *Li EPY. Sibling advocates of people with intellectual disabilities. Int J Rehabil Res. 2006;29(2):175-178. 16609332 10.1097/01.mrr.0000191845.65198.d7

[34] Baker BL Blacher J Pfeiffer B. Family involvement in residential treatment. Am J Ment. Retard. 1996;101:1-14. 8827247

[35] *Rimmerman A Muraver M. Undesired life events, life satisfaction and well-being of ageing mothers of adult offspring with intellectual disability living at home or out-of-home. J Intellect Dev Disabil. 2014;26(3):195-204.

[36] *Jansen S Van der Putten A Vlaskamp C. Parents’ experiences of collaborating with professionals in the support of their child with profound intellectual and multiple disabilities: a multiple case study. J Intellect Disabil. 2017;21(1):53-67. 27056641 10.1177/1744629516641843

[37] Schwartz C Tsumi A. Parental involvement in the residential care of persons with intellectual disability: the impacts of parents’ and residents’ characteristics and the process of relocation. J Appl Res Intellect Disabil. 2003;16:285-293.

[38] Griffith GM Hastings RP. ‘He’s hard work, but he’s worth it’. The experience of caregivers of individuals with intellectual disabilities and challenging behaviour: a meta-synthesis of qualitative research. J Appl Res Intellect Disabil. 2014;27:401-419. 24105755 10.1111/jar.12073

[39] Grey JM Totsika V Hastings RP. Physical and psychological health of family carers co-residing with an adult relative with intellectual disability. J Appl Res Intellect Disabil. 2018;31(Suppl. 2):191-202. 28378391 10.1111/jar.12353

[40] Turnbull A Turnbull R Erwin E. Families, Professionals, and Exceptionality. Positive Outcomes Through Partnerships and Trust. 7th ed. Pearson Academic; 2014.

